# Porous Glass Microspheres from Alkali-Activated Fiber Glass Waste

**DOI:** 10.3390/ma15031043

**Published:** 2022-01-28

**Authors:** Mokhtar Mahmoud, Jozef Kraxner, Hana Kaňková, Miroslava Hujová, Si Chen, Dušan Galusek, Enrico Bernardo

**Affiliations:** 1 FunGlass, Centre for Functional and Surface Functionalized Glass, Alexander Dubček University of Trenčín, 911 50 Trenčín, Slovakia; jozef.kraxner@tnuni.sk (J.K.); hana.kankova@tnuni.sk (H.K.); miroslava.hujova@tnuni.sk (M.H.); si.chen@tnuni.sk (S.C.); dusan.galusek@tnuni.sk (D.G.); 2 Department of Industrial Engineering, University of Padova, 35131 Padova, Italy; enrico.bernardo@unipd.it; 3 Joint Glass Centre of the IIC SAS, TnUAD and FChFT STU, 911 50 Trenčín, Slovakia

**Keywords:** up-cycling, glass waste, alkali activation, flame synthesis, porous glass microspheres

## Abstract

Fiber glass waste (FGW) was subjected to alkali activation in an aqueous solution with different concentrations of sodium/potassium hydroxide. The activated materials were fed into a methane–oxygen flame with a temperature of around 1600 °C. X-ray diffraction analysis confirmed the formation of several hydrated compounds, which decomposed upon flame synthesis, leading to porous glass microspheres (PGMs). Pore formation was favored by using highly concentrated activating alkali solutions. The highest homogeneity and yield of PGMs corresponded to the activation with 9 M KOH aqueous solution.

## 1. Introduction

Reusing glass waste can considerably decrease the demand for new raw materials for glass production and contribute to the reduction of landfills, known to represent a multiform ecological problem (in terms of consumption of free land, water and air pollution, and emission of greenhouse gases) [1,2].

Landfilling can be significantly reduced only if waste glass is involved in the manufacturing of a new generation of original glass articles. The sustainability of this approach (e.g., cullet from crushed container used for new containers) is complicated by the need for expensive sorting operations, aimed at separating glass from other materials, glasses of different chemical compositions, or even glasses with different colors [3]. Some materials, such as residues from the production of glass fibers (fiber glass waste, FGW), are practically ‘unrecyclable’ [4]. The utilization of FGW can be accomplished either by decreasing the cost of cleaning and sorting through applying new procedures or by applying an up-cycling approach, consisting of the obtainment of innovative products, which could justify the expense of processing [5].

Recent trends in glass up-cycling often employ alkali activation, i.e., the (at least) partial dissolution of glass in concentrated aqueous solutions of alkali hydroxides, aluminates, and silicates that yields hydrated compounds. When subjected to condensation reactions, they form a gel at room temperature or typically below 100 °C [4]. According to the specific chemistry of waste glass, a stable (alumino-silicate) ‘zeolite-like’ network, useful as a new cementitious material, may be formed. Glass fibers, in particular, may yield high-strength gels by reaction with Na-aluminate solutions [6]. The alkali activation may be used also as an intermediate step, since glass suspensions, before complete setting, may be foamed by intensive mechanical stirring, leading to highly porous bodies, later consolidated by sintering. When a stable gel is not the main objective, the glass can be simply activated by reaction with less expensive alkali hydroxides [7].

The present paper concerns the exploitation of alkali activation for the manufacturing of highly porous glass microspheres (PGMs), following a preliminary study on Bioglass^®^ 45S5 [8]. Powders prepared by the crushing of hardened suspensions are fed into a flame synthesis apparatus: glass microbeads formed by high-temperature processing are simultaneously expanded by gases released upon the decomposition of hydrated compounds [8]. PGMs derived from glass waste are optimized using different concentrations of alkaline solutions. The prepared PGMs could be applied as filtration materials or in hydrogen storage applications [9].

## 2. Materials and Methods

FGW (56.4 SiO_2_, 23.2 CaO, 13.6 Al_2_O_3_, 5.1 B_2_O_3_, 0.4 Na_2_O, 0.6 K_2_O, 0.4 TiO_2_, 0.2 Fe_2_O_3_ in wt.%, data obtained by XRF analysis) was crushed and a fraction below 40 µm was obtained by sieving. NaOH and KOH (reagent grade, Penta, Bratislava, Slovakia) were dissolved in deionized water to form activating solutions of different molarity (3 M, 5 M, 7 M, 9 M NaOH; 9 M 50 mol% NaOH—50 mol% KOH; 9 M KOH). Glass waste powders were suspended in the solutions with a solid loading of 65 wt.% by mechanical stirring (1 h, at 500 rpm). The suspensions were poured into polystyrene containers and left to dry at 75 °C for 24 h.

Fine powders obtained by crushing of hardened suspensions were characterized by X-ray diffraction (XRD-Panalytical Empyrean, CuKα radiation, λ = 1.5405 Å) and thermogravimetry (STA449 F1 Jupiter TG/DTA/DSC, Netzsch, Selb, Germany), using air, with a flow rate of 30 mL/min. Sieved powder (40–80 µm fraction) was then fed into a methane–oxygen flame at 1600 °C and glass microspheres were prepared by quenching the melt droplets with deionized water. The produced glass microspheres were collected in a sedimentation tank, filtered, and washed with deionized water.

The chemical composition of obtained glass microspheres was determined by ICP (ICP OES 5100 SVDV Agilent, Santa Clara, CA, USA), using a decomposition–microwave digestion system, speedwave 4 Berghof, 20 mg + 6 mL HCl+ 2 mLHNO3 + 0.5 mL HF. Nebulizer flow was 0.55 L/min and plasma flow was 12 L/min.

**Figure 1:**
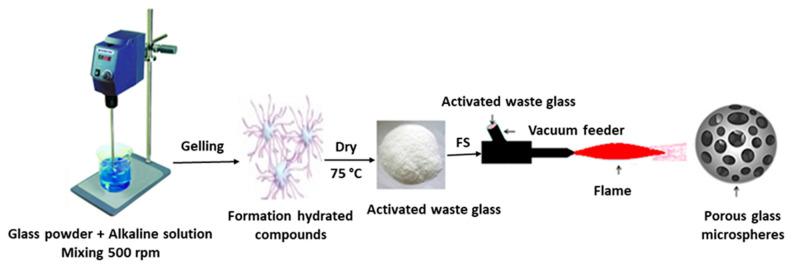
Scheme of production of PGMs according to alkali activation combined with flame synthesis.

SEM was used to study the morphology of prepared glass microspheres (JSM-7600F Schottky Field Emission Scanning Electron Microscope), applying normal operation, 1.2 kVA. A schematic drawing of the PGM production process by a combination of alkali activation and flame synthesis is shown in Figure 1.

## 3. Results and Discussion

**Figure 2:**
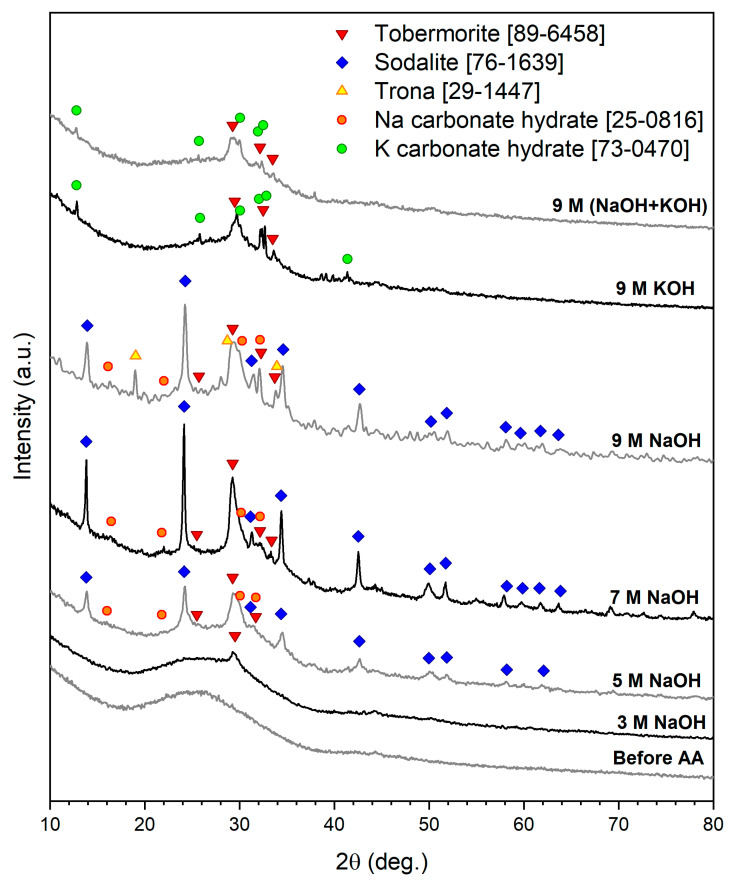
X-ray diffraction patterns of FGW before and after alkali activation.

The starting glass waste was X-ray amorphous, as shown in Figure 2. Glass matrix bonds including Si-O-Si, Al-O-Si, and Ca-O are attacked by the alkaline solutions during the alkali activation process, yielding hydrated compounds, which form coagulated structures, leading to condensation and crystallization [10,11]. The activation of glass waste with a relatively weak alkaline solution (3 M NaOH) promoted the formation of a calcium silicate hydrate (C-S-H) compound, (tobermorite A, Ca_5_(Si_6_O_16_)(OH)_2_, PDF 89-6458), which was in line with the results previously observed in [4].

The amount of the phase, belonging to so-called C-S-H compounds, typically formed upon the hydration of conventional cements, increased with increasing molarity of the alkaline activators [12,13]. By increasing the concentration to 5 M NaOH, tobermorite was accompanied by sodium alumino-silicate hydrate (N-A-S-H) (zeolite phase) (sodalite, 4Na_2_O·3Al_2_O_3_·6SiO_2_·3H_2_O, PDF 76-1639), as shown in Figure 2. This suggests the more extensive dissolution of the calcium alumino-silicate FGW matrix and a higher concentration of hydrated compounds (C-S-H and N-A-S-H); see Table 1.

Dissolution in 7 M NaOH led also to the development of hydrated sodium carbonate phases (Na_2_CO_3_·7H_2_O, PDF 25-0816, and Na_3_H(CO_3_)_2_·2H_2_O, PDF 29-1447—also known as ‘trona’). It was observed that the peak intensities reached their maximum by applying 7 M NaOH, which indicated the formation of the highest quantity of the hydrated compounds. Moreover, 9 M NaOH yielded the same hydrated compounds in lower quantities. At high alkali concentrations (<9 M NaOH), the silicic acid activity in the solution increased due to the congruent dissolution of the glass matrix. The chemical affinity for further dissolution is thus reduced. This means that less alkali is incorporated into the obtained glass [14].

Using concentrated alkaline solutions initiated and increased the incorporation of ionic species, which led to restricting the movement of ions and inhibited the polymerization process [15]. When the alkali activation occurs at a high temperature, the viscosity of the system will be higher, and the dissolution of silica will be lower, which leads to a lower contribution in the gel and weakness of the formed chain [16].

On the other hand, dissolution of FGW in 9 M KOH and 9 M (KOH+NaOH) suppressed the formation of Na-containing compounds, yielding only tobermorite and hydrated potassium carbonate. The hydroxide ions and potassium ions from KOH retarded the diffusion of NaOH through the solution; therefore, dissolution of the glass fibers required more activation energy [17].

**Figure 3:**
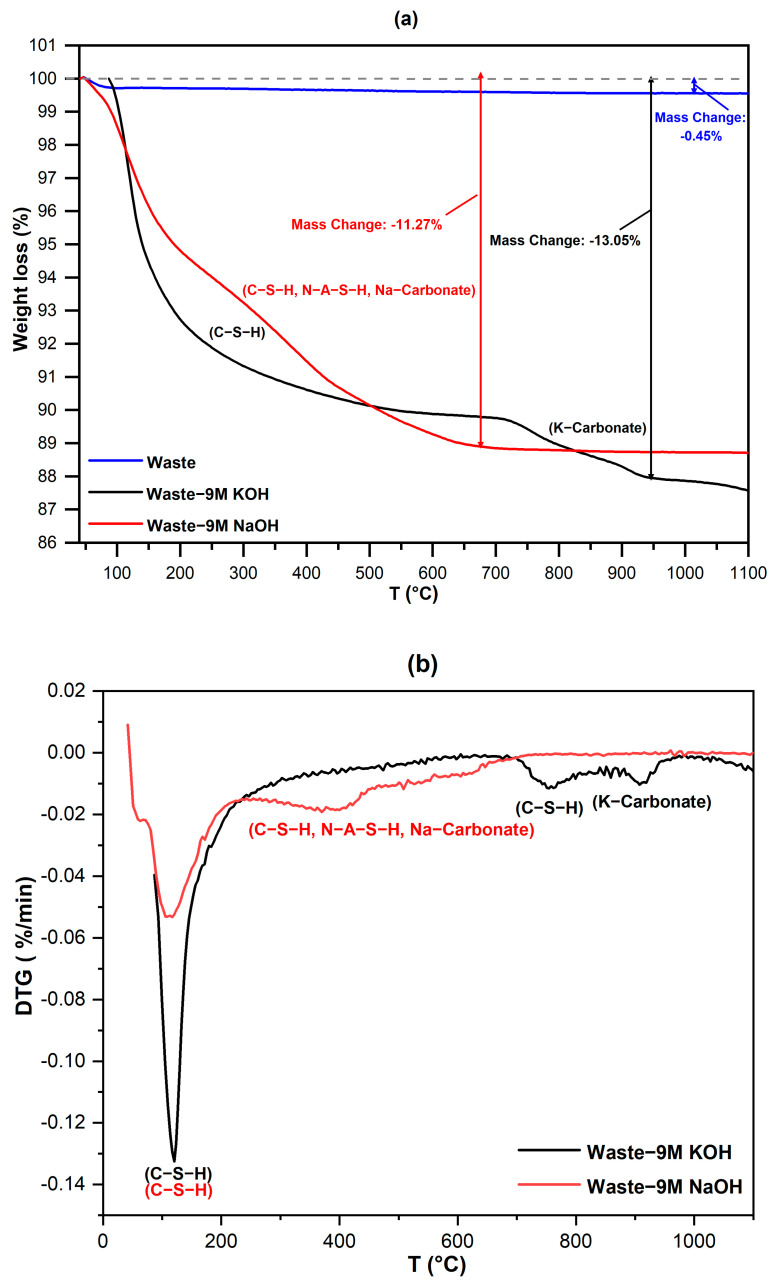
TG (**a**) and DTG (**b**) curves of activated glass waste.

C-S-H compounds are of particular interest since they are known to decompose completely at relatively high temperatures from 105 to 1000 °C [18]. Upon flame synthesis, porous microbeads are formed only if some gases are still released at the temperatures at which droplets of molten glass form. The results of thermogravimetric analysis (TG and DTG) revealed that the gas release was completed at approximately 600 °C in FGW activated by 9 M NaOH solution, where all the formed hydrated compounds (C-S-H, N-A-S-H, Na-carbonate) decomposed and evolved into gases in two steps, as shown in Figure 3a,b. The activation with KOH led to significant gas evolution from 800 to 950 °C. The decomposition of the hydrated compounds systematically followed three steps, as shown in Figure 3b. The first step started at around 120 °C, which indicated the degradation of C-S-H compounds, followed by the decomposition of the residual C-S-H compound at around 800 °C; then, the last step was dedicated to the decomposition of the K-carbonate hydrate phase at around 950 °C. In conclusion, the decomposition of the hydrated compounds near the melting point of glass resulted in porous glass microspheres [19]. Hence, it was confirmed that applying KOH as an alkaline activator could yield superior results regarding the formation of porous glass microspheres. The strategy to successfully produce a porous glass structure is to carefully choose the appropriate alkaline activators [20].

Flame synthesis did not only result in the softening of glass, but also mixed unreacted glass with the residues of the decomposition of the gels, incorporating volatile alkali oxides into the melt. As shown in Table 1, the chemical composition of the glass microspheres was altered. In practice, the activation resulted in two conflicting effects: highly concentrated alkali solutions promoted the formation of hydrated compounds responsible for the gas release, but the incorporation of alkalis reduced the viscosity of the melt during flame synthesis, causing the collapse of cellular structures.

**Figure 4:**
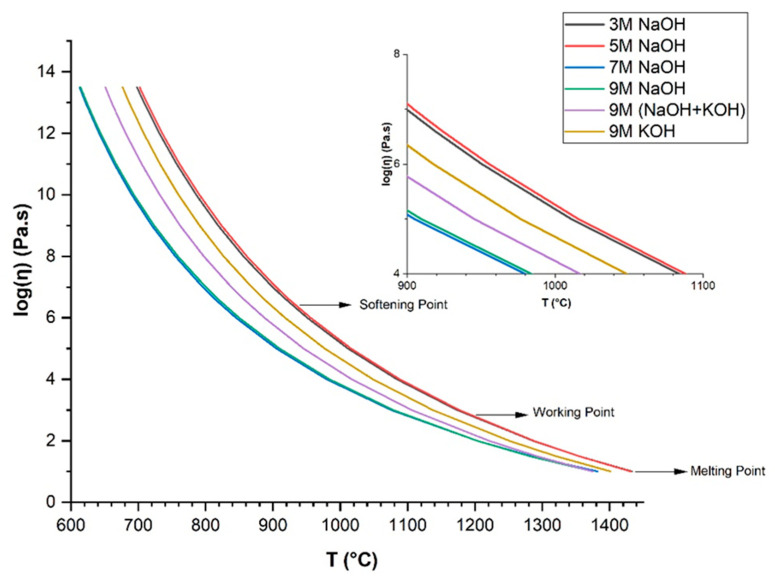
Viscosity–temperature curves of the investigated glass compositions calculated from the measured glass composition with the use of the VFT equation.

The viscosity–temperature relation in glasses was calculated by the Vogel–Fulcher–Tammann (VFT) equation: log(η) = A + B/(T − T_0_) [21], where A is the logarithm of the viscosity at the high temperature, and B is the free fitting parameter of the VFT equation. Based on the chemical composition of glasses (activated by different concentrations of alkaline solutions, Table 1), the viscosity–temperature relation of the batch can be estimated, as shown in Figure 4. The increase in Na_2_O content in the glasses activated by 3–7 M NaOH solutions decreased the viscosity in the glass melting range, while activation with 9 M NaOH led to a glass with slightly lower Na_2_O content and moderately higher viscosity.

Mixed alkalis (Na_2_O and K_2_O) shifted the viscosity/temperature curve close to those for glasses activated by NaOH, due to alkali–alkali interactions; the mixed alkali effect is a manifestation of a general nonlinear composition–viscosity behavior [22]. Activation with pure KOH increased the content of K_2_O in the glass and shifted the viscosity curve to moderately lower values. The results correspond to the study of Isard, who demonstrated that alkali and alkaline earth oxides decreased the viscosity in glass melts in the approximate order MgO < CaO < SrO < BaO < K_2_O < Na_2_O [23]. The alkaline earth oxides possess the ability to bridge over two non-bridging oxygen sites at low temperatures, therefore strengthening the network and increasing the viscosity [24].

**Figure 5:**
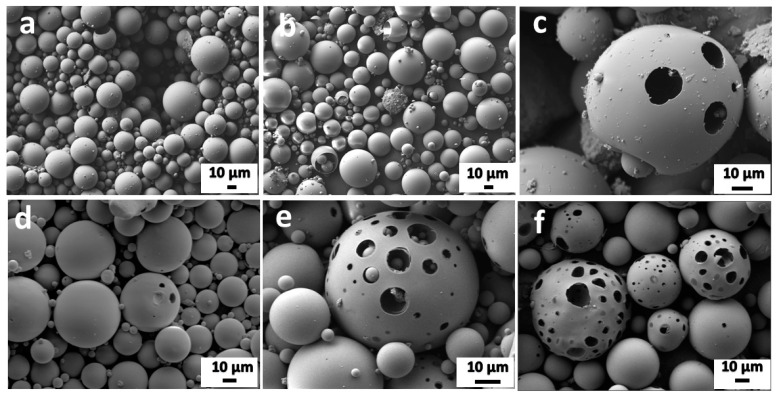
SEM micrographs of glass microspheres after alkali activation by (**a**) 3 M NaOH, (**b**) 5 M NaOH, (**c**) 7 M NaOH, (**d**) 9 M NaOH, (**e**) 9 M (NaOH + KOH), (**f**) 9 M KOH.

The SEM micrographs show the formation of solid microspheres from glass activated with 3 M NaOH solution (Figure 5a), where FGW is in 3 M NaOH dissolved to a comparatively lower extent, as supported by the XRD results (Figure 2). Increasing the hydrated compounds slightly using 5 M NaOH activator is not sufficient to induce pores, as revealed in Figure 5b, whereas applying effective concentrated solutions (7 and 9 M NaOH) leads to the formation of porous glass microspheres. With the increasing concentration of NaOH, the amount of hydrated compounds is increased; hence, a higher quantity of porogen gases is released upon decomposition, ultimately leading to the formation of larger pores [25]. However, it is important to note that the overall quantity of pores is still limited (Figure 5c,d). The application of a mixed alkali solution (50 mol% KOH + 50 mol% NaOH) seemingly enhanced the quantity of the formed pores (Figure 5e). This might be due to the formation of the K-carbonate hydrate phase (Figure 2), which decomposes at relatively high temperatures (Figure 3b). Finally, increasing the amount of K-carbonate hydrate phase under 9 M KOH alkaline activation seems to yield the highest amount of pores in glass microspheres (Figure 5f).

## 4. Conclusions

The impact of alkali activation using different concentrations of alkaline solutions on the preparation of porous glass microspheres from fiber glass waste was studied. Lower concentrations of alkaline solution yielded solid glass microspheres, which means that alkali activation under 7 M NaOH does not produce a sufficient amount of hydrated phases necessary for pore formation. Moreover, 7 M and 9 M NaOH solutions initiated the formation of porous glass microspheres. The mixed 9 M alkali solution (50 mol% KOH + 50 mol% NaOH) furthermore seemingly improved the quantity of pores. The most positive results were yielded with 9 M KOH solution, where we observed the highest quantity of pores in the glass microspheres.

## Figures and Tables

**Table 1:** Chemical composition of the obtained glass microspheres (% Wt.).

Glass Microspheres	Oxides Ratio (Wt.%)							
	SiO_2_	CaO	Al_2_O_3_	B_2_O_3_	Na_2_O	K_2_O	TiO_2_	Fe_2_O_3_
FGW + 3 M NaOH	61 ±3	24 ±2	13 ±1	0.40 ±0.01	0.1 ±0.02	–	0.50 ±0.02	0.30 ±0.03
FGW + 5 M NaOH	60 ±1	24.2 ±0.7	13.7 ±0.6	–	0.70 ±0.01	–	0.50 ±0.01	0.30 ±0.03
FGW + 7 M NaOH	58 ±2	19.8 ±0.5	11.0 ±0.6	1.8 ±0.1	7.8 ±0.2	0.20 ±0.02	0.40 ±0.01	0.30 ±0.01
FGW + 9 M NaOH	58 ±2	19.5 ±0.5	11.1 ±0.5	3.2 ±0.2	6.5 ±0.1	0.40 ±0.02	0.40 ±0.01	0.30 ±0.01
FGW+ 9 M NaOH + KOH	57 ±2	19.8 ±0.4	12.0 ±0.6	2.6 ±0.2	3.0 ±0.1	4.3 ±0.1	0.40 ±0.01	0.30 ±0.01
FGW + 9 M KOH	59 ±2	19.6 ±0.9	11.6 ±0.9	1.8 ±0.1	0.30 ±0.02	5.9 ±0.1	0.40 ±0.01	0.30 ±0.02

## Data Availability

The data presented in this study are available on request from the corresponding author.
